# Supplementary material for: Many foliar endophytic fungi of Quercus gambelii are capable of psychrotolerant saprotrophic growth
Source: PLoS One. 2022 Oct 12;17(10):e0275845. doi: 10.1371/journal.pone.0275845 (PMC9555652; doi:10.1371/journal.pone.0275845)
Supplement: S1 Table — (DOCX) [file pone.0275845.s001.docx]

**S2 Table. Isolate designation and species identity.**

| Isolate name | Taxon | UNITE ID# | Collection site |
| --- | --- | --- | --- |
| QG2.9 | *Ophiognomonia* sp. | [SH1776477.08FU](https://unite.ut.ee/bl_forw_sh.php?sh_name=SH1776477.08FU) | Devil’s Kitchen |
| QG3.3.A | *Cladosporium herbarum* | [SH1744611.08FU](https://unite.ut.ee/bl_forw_sh.php?sh_name=SH1744611.08FU) | Devil’s Kitchen |
| QG3.6.A.1 | *Ophiognomonia* sp. | [SH1776477.08FU](https://unite.ut.ee/bl_forw_sh.php?sh_name=SH1776477.08FU) | Devil’s Kitchen |
| QG4.1.R1 | *Ophiognomonia setacea* | [SH1776430.08FU](https://unite.ut.ee/bl_forw_sh.php?sh_name=SH1776430.08FU) | Devil’s Kitchen |
| QG5.12.A.1 | *Tricharina cretea* | [SH1707615.08FU](https://unite.ut.ee/bl_forw_sh.php?sh_name=SH1707615.08FU) | Devil’s Kitchen |
| QG5.4.A | *Ophiognomonia* sp. | [SH1776477.08FU](https://unite.ut.ee/bl_forw_sh.php?sh_name=SH1776477.08FU) | Devil’s Kitchen |
| QG5.8.A.2 | Dothideales |  | Devil’s Kitchen |
| QG5.9.A | *Ophiognomonia* sp. | [SH1776477.08FU](https://unite.ut.ee/bl_forw_sh.php?sh_name=SH1776477.08FU) | Devil’s Kitchen |
| QG6.6.A.1 | *Ophiognomonia* sp. | [SH1776477.08FU](https://unite.ut.ee/bl_forw_sh.php?sh_name=SH1776477.08FU) | Devil’s Kitchen |
| QG6.7.A | *Cladosporium herbarum* | [SH1744611.08FU](https://unite.ut.ee/bl_forw_sh.php?sh_name=SH1744611.08FU) | Devil’s Kitchen |
| QG7.10 | *Ophiognomonia* sp. | [SH1776477.08FU](https://unite.ut.ee/bl_forw_sh.php?sh_name=SH1776477.08FU) | Devil’s Kitchen |
| QG7.10.A | Pezizomycetes | [SH1668522.08FU](https://unite.ut.ee/bl_forw_sh.php?sh_name=SH1668522.08FU) | Devil’s Kitchen |
| QG7.10.A.3 | *Cladosporium herbarum* | [SH1744611.08FU](https://unite.ut.ee/bl_forw_sh.php?sh_name=SH1744611.08FU) | Devil’s Kitchen |
| QGshd1.10.1 | *Ophiognomonia* sp. | [SH1776477.08FU](https://unite.ut.ee/bl_forw_sh.php?sh_name=SH1776477.08FU) | Slate Canyon |
| QGshd1.7.A.4.1 | *Ophiognomonia* sp. | [SH1776477.08FU](https://unite.ut.ee/bl_forw_sh.php?sh_name=SH1776477.08FU) | Slate Canyon |
| QGshd1.7.A.4.2 | *Ophiognomonia* sp. | [SH1776477.08FU](https://unite.ut.ee/bl_forw_sh.php?sh_name=SH1776477.08FU) | Slate Canyon |
| QGshd1.9.A.R2 | *Cladosporium allicinum* | [SH1744655.08FU](https://unite.ut.ee/bl_forw_sh.php?sh_name=SH1744655.08FU) | Slate Canyon |
| QGshd2.3.A.2 | *Ophiognomonia setacea* | [SH1776430.08FU](https://unite.ut.ee/bl_forw_sh.php?sh_name=SH1776430.08FU) | Slate Canyon |
| QGshd3.4.A.1 | *Ophiognomonia* sp. | [SH1776477.08FU](https://unite.ut.ee/bl_forw_sh.php?sh_name=SH1776477.08FU) | Slate Canyon |
| QGshd3.6.2 | *Apiognomonia errabunda* | [SH1776425.08FU](https://unite.ut.ee/bl_forw_sh.php?sh_name=SH1776425.08FU) | Slate Canyon |
| QGshd3.9.A.2 | *Apiognomonia errabunda* | [SH1776425.08FU](https://unite.ut.ee/bl_forw_sh.php?sh_name=SH1776425.08FU) | Slate Canyon |
| QGshd5.3.A.1 | Diaporthales |  | Slate Canyon |
| QGshd5.9.A.1 | *Ophiognomonia* sp. | [SH1776430.08FU](https://unite.ut.ee/bl_forw_sh.php?sh_name=SH1776430.08FU) | Slate Canyon |
| QGsun1.6.A.2 | *Ophiognomonia* sp. | [SH1776430.08FU](https://unite.ut.ee/bl_forw_sh.php?sh_name=SH1776430.08FU) | Slate Canyon |
| QGsun1.8.A.1.1 | Pezizomycetes |  | Slate Canyon |
| QGsun2.2.A | *Ophiognomonia setacea* | [SH1776430.08FU](https://unite.ut.ee/bl_forw_sh.php?sh_name=SH1776430.08FU) | Slate Canyon |
| QGsun2.2.A.1 | *Ophiognomonia setacea* | [SH1776430.08FU](https://unite.ut.ee/bl_forw_sh.php?sh_name=SH1776430.08FU) | Slate Canyon |
| QGsun2.9.A.3 | *Ophiognomonia setacea* | [SH1776430.08FU](https://unite.ut.ee/bl_forw_sh.php?sh_name=SH1776430.08FU) | Slate Canyon |
| QGsun3.10.2 | *Ophiognomonia* sp. | [SH1776430.08FU](https://unite.ut.ee/bl_forw_sh.php?sh_name=SH1776430.08FU) | Slate Canyon |
| QGsun3.10.A | *Coniochaeta* sp. | SH1837170.08FU | Slate Canyon |
| QGsun4.2.A | *Venturia sp.* |  | Slate Canyon |
| QGsun4.5.A.1 | *Apiognomonia errabunda* | [SH1776425.08FU](https://unite.ut.ee/bl_forw_sh.php?sh_name=SH1776425.08FU) | Slate Canyon |
| QGsun4.9.A.2.1 | *Pyronema omphalodes* | [SH1707606.08FU](https://unite.ut.ee/bl_forw_sh.php?sh_name=SH1707606.08FU) | Slate Canyon |
| QGsun5.2 | *Parafenestella sp.* |  | Slate Canyon |
| QGsun5.3.A | *Ophiognomonia setacea* | [SH1776430.08FU](https://unite.ut.ee/bl_forw_sh.php?sh_name=SH1776430.08FU) | Slate Canyon |
| QGsun5.5.A.2.2 | *Ophiognomonia* sp. | [SH1776430.08FU](https://unite.ut.ee/bl_forw_sh.php?sh_name=SH1776430.08FU) | Slate Canyon |
| QGsun5.7.A.4 | *Coniochaetales* |  | Slate Canyon |
| QGsun6.6.A | *Coniochaeta polymorpha* | [SH1837180.08FU](https://unite.ut.ee/bl_forw_sh.php?sh_name=SH1837180.08FU) | Slate Canyon |
| QGsun6.8 | *Apiognomonia errabunda* | [SH1776425.08FU](https://unite.ut.ee/bl_forw_sh.php?sh_name=SH1776425.08FU) | Slate Canyon |
| QGsun6.9 | *Ophiognomonia* sp. | [SH1776430.08FU](https://unite.ut.ee/bl_forw_sh.php?sh_name=SH1776430.08FU) | Slate Canyon |
