# Supplementary material for: Many foliar endophytic fungi of Quercus gambelii are capable of psychrotolerant saprotrophic growth
Source: PLoS One. 2022 Oct 12;17(10):e0275845. doi: 10.1371/journal.pone.0275845 (PMC9555652; doi:10.1371/journal.pone.0275845)
Supplement: S1 File — (DOCX) [file pone.0275845.s002.docx]

##### **S1 Bootstrapping R script**

install.packages("boot",dep=TRUE)

library(boot)

treatment.vec = c(#growth rates of all 4 replicates of one isolate grown on leaf litter medium)

glucose.vec = c(#growth rates of all 4 replicates of isolate grown on glucose medium)

treatment.vec

glucose.vec

combinations = expand.grid(treatment.vec,glucose.vec)

combinations

treatment.comb = combinations[c(1:1)]

glucose.comb = combinations[c(2:2)]

treatment.comb

glucose.comb

difs = treatment.comb-glucose.comb

difs.vec = difs[,1]

BootstrapMean = function(X=difs.vec){

x.boot=sample(X, size=4, replace=T)

mean(x.boot) }

N = 1000

boot.replicate = replicate(1000, BootstrapMean() )

stat = rep(NA, N)

for (i in 1:N){

stat[i] = BootstrapMean()}

par(mfrow=c(1,2))

hist(stat)

abline(v=mean(difs.vec), lwd=2, col = "red")

hist(boot.replicate)

abline(v=mean(difs.vec), lwd=2, col = "red")

BootstrapMean()

mean(difs.vec)

mean = mean(stat)

mean

stddev = sd(stat)

SE = (sd(stat)/sqrt(4))

SE

error **=** qnorm(0.975)*****stddev**/**sqrt(4)

left **=** mean**-**error

right = mean+error

list (mean, stddev, left, right)
