# Supplementary material for: Many foliar endophytic fungi of Quercus gambelii are capable of psychrotolerant saprotrophic growth
Source: PLoS One. 2022 Oct 12;17(10):e0275845. doi: 10.1371/journal.pone.0275845 (PMC9555652; doi:10.1371/journal.pone.0275845)
Supplement: Attachment — Submitted filename: Response to reviews PLOS one.docx [file pone.0275845.s004.docx]

25 April 2022

We thank the editor, Raffaella Balestrini, for the opportunity to resubmit this manuscript (PONE-D-21-38995).

Roger Koide

Reviewers' comments:

Reviewer's Responses to Questions

**Comments to the Author**

Reviewer #1: Dear authors,
I read your article with interest and I found it inspiring, I personally be sure that this kind of study are still important, also in the omic-era, where powerful tools are available to study many different microorganisms species contemporary, but on the other side it became difficult to really understand who-do-what.

Your study on psychrophilic endophytic fungi isolated from Quercus gambelii leaves try to verify two interesting hypothesis on fungal ability to be active in cool environment and the tradeoff activity of these fungi between winter and summer seasons. The approach that you have used is interesting, but in my opinion it lacks some tricks that would make this work much more incisive, at present I do not think it is publishable, because it has too many weak points; furthermore, the policy of the journal is to favour studies with innovative approaches and in your case innovations are not adequately described and justified. I am sorry, I will not recommend the publication of your study, BUT in order to give my contribution I would report my main observations below:

Psychrotolerance has been documented among endophytic fungi only twice before, but on both occasions the experiments were performed with a sugar as the carbon source. Psychrotolerant **saprotrophy** has never been previously documented among endophytic fungi from any system. Therefore, we feel that our study is innovative. This point is now clearer throughout the manuscript.

- Quercus gambelii is not only found in Utah, but also (at least) in Colorado, New Mexico and Arizona. Your work is Utah-centric, but the world is full of arid places with rainy winters, try to extend your discussion to all these environments (the Mediterranean is an example), it would certainly be more useful and interesting.

We have attempted to make the manuscript less Utah-centric, particularly in the introduction (first paragraph) and materials and methods (first paragraph). We note, however, that the combination of moisture availability mainly in the cold months of the year and the presence of *Quercus gambelii* is not found in many parts of the world. Moreover, we have heeded the caution given by the journal: “submissions will be rejected if the interpretation of results is unjustified or inappropriate so authors should avoid overstating conclusions”. Therefore, we did not want to interpret our work too broadly.

- The discussion is very poor, many of your statements, not supported by adequate citations are very very strong, but without a counterpart in the results.

The discussion has been extensively rewritten to eliminate unwarranted statements and to accurately portray what we do not yet know.

- I am not at all surprised that most species are cold tolerant (psychrophilic, I guess is the most correct term) ... after all they are species that live in this environment!

The reviewer expresses no particular surprise that we found psychrotolerance among our fungal isolates. We wish to make two points here. First, there is no *a priori* reason to expect endophytic fungi to be psychrotolerant. If endophytic fungi were strictly biotrophic (as opposed to saprotrophic), they would only have to maintain activity during the growing season, which does not include winter and early spring.

Second, psychrotolerance has been noted among endophytic fungi growing twice previously (cited in the manuscript) but, as we note in the manuscript, in both cases the fungi were grown on a glucose-based medium (not saprotrophic growth). Psychrotolerance is of ecological interest primarily in the context of saprotrophy because litter is available as a carbon source only when moisture is available for decomposition, which is mainly in the coldest months of the year.

For a better understanding of the effects of cold and moisture, I recommend that you extend your sampling to areas where environmental conditions are less severe and/or in contrast. You can isolate fungi from different environments and then grow them on different substrates and cross conditions.

In our manuscript we asked the simple but *novel* question as to whether endophytic fungi can be psychrotolerant during a saprotrophic existence. This reviewer suggests we ask a different question, which is whether fungi from different habitats differ in the frequency of psychrotolerance. That is a very good question, indeed, with tremendous ecological relevance, but it is not the question we posed. It does not seem fair to fault our study because we asked a question different from the one this reviewer wishes we asked.

- You isolated fungi on a single medium (MEA 2%): multiplying the media and varying the concentrations is a great way to isolate more taxa.

Yes, we might possibly have obtained more isolates using different types of media. We undoubtedly did not isolate all that were present, but we do not suppose that such is possible no matter how many media were used. Nevertheless, we obtained a total of 675 isolates using the one type of medium. From the 40 isolates we ended up testing (see manuscript for selection criteria), we found that a large majority (36) were capable of psychrotolerant saprotrophy and that a few were not. Our data, therefore, were sufficient to test our hypotheses.

The isolation was only done at 17°C, I would have expected it to be done at 5°C as well.

The materials and methods now explain how we isolated our fungi in greater detail. Briefly, we initially isolated fungi at 10°C as recommended in a previous study of psychrotolerance. This procedure resulted in very few isolates. We subsequently incubated the leaf disks at 17°C and isolated additional fungi, many of which were psychrotolerant despite being isolated at 17°C. In other words, the relatively warm temperature of isolation did not prevent psychrotolerant species from being isolated. Indeed, in testing hypothesis 2 we found a **positive** correlation between growth at 5°C and 17°C.

- The correlation between growth and activity may be right, but the statistics fielded are too weak to verify it.

The “activity” we measured was growth. Therefore, we have eliminated the term “activity” and, instead, use “growth”. We also more fully explain the statistical methods we used to analyze saprotrophic capacity, particularly the need for calculating the error by bootstrapping and the somewhat unconventional analysis of variance necessitated by bootstrapping.

- You did not extract the DNA, but went straight into PCR, with a 15 minute long first denaturation step. This is a method I would use for bacteria, but fungi, they have complex walls and huge enzyme machinery. No wonder you had to lower the sequence quality standard to 10! However, in this way the sequences are not really reliable. Where did you find this method?

Many parts of the materials and methods section have been rewritten to improve clarity. In particular, the section on PCR now more fully justifies the use of direct PCR for fungi by citing three of many publications using direct PCR of fungal colonies:

Walch G, Knapp M, Rainer G, Peintner U. Colony-PCR is a rapid method for DNA amplification of hyphomycetes. J Fungi. 2016;2. doi:10.3390/jof2020012

Varga I, Poczai P, Cernák I, Hyvönen J. Application of direct PCR in rapid rDNA ITS haplotype determination of the hyperparasitic fungus Sphaeropsis visci (Botryosphaeriaceae). J Korean Phys Soc. 2014;3: 1–9. doi:10.1186/2193-1801-3-569

AlShahni MM, Makimura K, Yamada T, Satoh K, Ishihara Y, Takatori K, et al. Direct colony PCR of several medically important fungi using Ampdirect® Plus. Jpn J Infect Dis. 2009;62: 164–167.

- You say you don't know which portion of the fungi you have isolated: with a quick metabarcoding essay you can easily find it out, the costs are now very low and the bio-informatics child-proof.

Actually, high throughput sequencing is not really capable of determining the proportion of endophytic fungi that we were able to isolate. The difficulty is that any isolate with the same ITS sequence will register as the same species. However, it is becoming clear that different isolates of a given species may be functionally unique. Therefore, this approach will not necessarily give us a good idea of how many distinct isolates exist in plant tissues. We have consequently dropped any discussion of the commonality of psychrotolerance among endophytic fungi of *Q. gambelii.* Thinking about these issues gave us an opportunity to reorganize the discussion, which has markedly improved its clarity.

- There are many types of enzymatic assays with which to characterise the activity of microorganisms in a substrate, similarly with RT-PCR it is possible to target the activity of a single species for a single enzyme. CAZymes, Peroxidases, ... are all very important players in this context ... you didn't even mention them in the introduction or discussion. My advice,ultimately, is to expand the experiment and resubmit it, in a more robust way to the journal.

Yes, enzymes are definitely used by saprotrophic fungi to oxidize and hydrolyze litter materials. However, in this study we did not ask whether any of these enzymes were involved. One might say our study was largely phenomenological rather than mechanistic. We posed two simple hypotheses and we focused our efforts on testing them. Studying the enzymes, perhaps, would be a good next step, but it was not a part of our study. Again, it does not seem fair to fault our study because we asked questions that were different from the one this reviewer wishes we asked.
