# Supplementary material for: Many foliar endophytic fungi of Quercus gambelii are capable of psychrotolerant saprotrophic growth
Source: PLoS One. 2022 Oct 12;17(10):e0275845. doi: 10.1371/journal.pone.0275845 (PMC9555652; doi:10.1371/journal.pone.0275845)
Supplement: Attachment — Submitted filename: Response to reviewers.docx [file pone.0275845.s005.docx]

Response to reviewers

If you would like to make changes to your financial disclosure, please include your updated statement in your cover letter. Guidelines for resubmitting your figure files are available below the reviewer comments at the end of this letter.

**There is no need to make changes to the financial disclosure statement. Thank you.**

Journal Requirements:

When submitting your revision, we need you to address these additional requirements.

1. Please ensure that your manuscript meets PLOS ONE's style requirements, including those for file naming. The PLOS ONE style templates can be found at

<https://journals.plos.org/plosone/s/file?id=wjVg/PLOSOne_formatting_sample_main_body.pdf> and

<https://journals.plos.org/plosone/s/file?id=ba62/PLOSOne_formatting_sample_title_authors_affiliations.pdf>

**I have made the prescribed changes to the manuscript.**

2. In your Methods section, please provide additional information regarding the permits you obtained for the work. Please ensure you have included the full name of the authority that approved the field site access and, if no permits were required, a brief statement explaining why.

**This statement is now included in the methods section.**

3. Thank you for stating the following in the Acknowledgments Section of your manuscript:

“The authors acknowledge financial support from the Roger Sant Foundation and the Department of Biology, Brigham Young University.”

We note that you have provided additional information within the Acknowledgements Section that is not currently declared in your Funding Statement. Please note that funding information should not appear in the Acknowledgments section or other areas of your manuscript. We will only publish funding information present in the Funding Statement section of the online submission form.

Please remove any funding-related text from the manuscript and let us know how you would like to update your Funding Statement. Currently, your Funding Statement reads as follows:

“The authors acknowledge financial support from the Roger Sant Foundation and the Department of Biology, Brigham Young University.”

**The funding statement is sufficient, and I have eliminated the acknowledgement.**

Please include your amended statements within your cover letter; we will change the online submission form on your behalf.

**There is no need to make changes to the funding statement. Thank you.**

4. We note that you have indicated that data from this study are available upon request. PLOS only allows data to be available upon request if there are legal or ethical restrictions on sharing data publicly. For more information on unacceptable data access restrictions, please see <http://journals.plos.org/plosone/s/data-availability#loc-unacceptable-data-access-restrictions>.

In your revised cover letter, please address the following prompts:

a) If there are ethical or legal restrictions on sharing a de-identified data set, please explain them in detail (e.g., data contain potentially sensitive information, data are owned by a third-party organization, etc.) and who has imposed them (e.g., an ethics committee). Please also provide contact information for a data access committee, ethics committee, or other institutional body to which data requests may be sent.

b) If there are no restrictions, please upload the minimal anonymized data set necessary to replicate your study findings as either Supporting Information files or to a stable, public repository and provide us with the relevant URLs, DOIs, or accession numbers. For a list of acceptable repositories, please see <http://journals.plos.org/plosone/s/data-availability#loc-recommended-repositories>.

We will update your Data Availability statement on your behalf to reflect the information you provide.

**The data set is now provided as a supporting information file (S3_File).**

5. Please include captions for your Supporting Information files at the end of your manuscript, and update any in-text citations to match accordingly. Please see our Supporting Information guidelines for more information: <http://journals.plos.org/plosone/s/supporting-information>.

**Captions for the Supporting Information files** **have now been added to the manuscript.**

6. Please review your reference list to ensure that it is complete and correct. If you have cited papers that have been retracted, please include the rationale for doing so in the manuscript text, or remove these references and replace them with relevant current references. Any changes to the reference list should be mentioned in the rebuttal letter that accompanies your revised manuscript. If you need to cite a retracted article, indicate the article’s retracted status in the References list and also include a citation and full reference for the retraction notice.

**The reference list has been updated.**

Reviewers' comments:

Reviewer's Responses to Questions

Comments to the Author

1. Is the manuscript technically sound, and do the data support the conclusions?

The manuscript must describe a technically sound piece of scientific research with data that supports the conclusions. Experiments must have been conducted rigorously, with appropriate controls, replication, and sample sizes. The conclusions must be drawn appropriately based on the data presented.

Reviewer #1: Yes

Reviewer #2: Yes

2. Has the statistical analysis been performed appropriately and rigorously?

Reviewer #1: Yes

Reviewer #2: Yes

3. Have the authors made all data underlying the findings in their manuscript fully available?

The [PLOS Data policy](http://www.plosone.org/static/policies.action#sharing) requires authors to make all data underlying the findings described in their manuscript fully available without restriction, with rare exception (please refer to the Data Availability Statement in the manuscript PDF file). The data should be provided as part of the manuscript or its supporting information, or deposited to a public repository. For example, in addition to summary statistics, the data points behind means, medians and variance measures should be available. If there are restrictions on publicly sharing data—e.g. participant privacy or use of data from a third party—those must be specified.

Reviewer #1: Yes

Reviewer #2: No. **The dataset is now available as a supplemental file.**

4. Is the manuscript presented in an intelligible fashion and written in standard English?

PLOS ONE does not copyedit accepted manuscripts, so the language in submitted articles must be clear, correct, and unambiguous. Any typographical or grammatical errors should be corrected at revision, so please note any specific errors here.

Reviewer #1: Yes

Reviewer #2: Yes

5. Review Comments to the Author

Please use the space provided to explain your answers to the questions above. You may also include additional comments for the author, including concerns about dual publication, research ethics, or publication ethics. (Please upload your review as an attachment if it exceeds 20,000 characters)

Reviewer #1: The paper is well written, well organized, and absolutely clear about how the authors conducted the research. The study could be of certain interest to the target of Plos One.
Nevertheless, the overall quality of the manuscript needs to be slightly improved before publishing in Plos One, since minor issues are present.
I suggest the authors to better explain the second hypothesis in the material and method section since just a few words were spent about the goal of this second part of the paper.

**The second hypothesis is given in the introduction. It is a straightforward hypothesis about an ecological tradeoff, and other examples of such tradeoffs are given in the introduction for clarification. Therefore, there doesn’t seem to be a need to further explain this hypothesis in the materials and methods section, which is devoted only to explaining the methods used to test the hypothesis.**

Moreover, it is important to improve the clarity and quality of the tables provided in the text deriving from ANOVA analyses; it is not specified the number of ways (one way or two way) as well as the meaning of the acronyms.

**The structure of the tables indicates that both anovas were one-way analyses. I am not sure what the reviewer means as far “acronyms” are concerned. The “df”, “SS”, “MS”, “F” and “P” are straightforward abbreviations of well-known anova terms (degrees of freedom, sums of squares, mean squares, F statistic and P value). If a reader does not recognize these terms, they will not be able to decipher an anova table anyway.**

Furthermore, It would be useful to provide more details on the result of the statistical analysis reported in the tables within the text. Which variance gave greater significance? Between groups or within groups? This is important since It seems that these parameters differ a lot in terms of explained variance.

**The variance is given by the sums of squares. The very reason we included the complete anova table was to allow the reader to make this assessment. Indeed, in the results section, we already make statements such as “with among species variation accounting for 39% of the total variability”.**

Minor issues
The figures contains a lot of data, but are not properly legible. Is it possible to rotate the figures? I understand that could negatively affect the figure quality, but It surely could improve from a legibility point of view.

**Yes, the figures could be rotated, but I do not see how that would increase how easily the figures are understood. Species names, some of which are very long, are not given in these figures to keep the figure as simple and easily interpreted as possible. S2 Table lists the species name for each of the isolates.**

Reviewer #2: I think the authors satisfactorily replied to the reviewer 1 which raise some issues that go beyond the aim of the work. Moreover, some comments are inappropriate (e.g., since mid-2000s direct PCR is successfully applied to fungal material without any problem about the sequence quality). The aim of the work is clear and the biological hypotheses which the work is based on are appropriate and fully met. Experimental planning and procedures are detailed and they support appropriately what the authors intended to demonstrate although some parts of the M&M section can be improved (see the below comments).

**Thank you for that confirmation.**

However, it is not clear because you selected as “warm” temperature 17 °C that in many parts of the world is not so “warm”. I think this has to do with the climate where Q. gambelii grow. In this case the choice of this temperature level should be justified and an in-depth description of the study site should be reported (e.g., min and max temperatures of coldest and warmest months).

**We now indicate in the materials and methods section that “air temperatures were monitored at the Devil’s Kitchen site from 24 Jun 2019 to 24 Sep 2019 using a temperature data logger (HOBO UA-001-64, Onset Computer Corporation, Bourne, MA, USA), set to log hourly.” Furthermore, in the section “Testing hypothesis 1: psychrotolerance”, we added “Each of the 40 isolates was grown in a 2 x 2 factorial experiment with two incubation temperatures (5 and 17 ºC) and two media (control and leaf litter). Our criterion for psychrotolerance was statistically significant growth at 5 ºC. The warmer temperature (17 ºC) represents the temperature during the growing season for *Q. gambelii*. The actual daily average temperature at Devil’s Kitchen during the majority of the growing season (24 Jun 2019 to 24 Sep 2019) was 16.9 °C (SD, 6.2).”**

The results about strain identification (ln 114-120) should be moved at the beginning of the result section and the phylogenetic tree generated to identify the unknown isolates should be shown. This part is a result and not a methodology and, in addition, this shift would compensate the gap of length between M&M and Result sections.

**As suggested, the identity of the isolates has been moved to the results section. However, we consider the generation of the phylogenetic tree to be part of the methods section because it was used to identify five of the unknown isolates. Therefore, the section title for this part of the materials and methods has been changed to “Sequencing and assigning taxonomy of fungal isolates”. Because the tree was used as a method and was not a result, we do not feel it should be included as a result.**

Accession numbers and site of collection of each isolate should be added in the supplementary Table 1

**The UNITE ID numbers and collection sites have been added to S2 Table.**

Specific comments
Ln 40, a brief description of the differences between psychrotolerant, psychrotrophic and psychrophilic should be provided to ensure that the reader understand why you chose the first term.

**Unfortunately, these terms have been used interchangeably depending on the author. Therefore, any of the terms could have been used. We chose psychrotolerant because the condition of a psychrotolerant organism is easily described as “psychrotolerance”, whereas the conditions describing a psychrotrophic or psychrophilic organism are awkward.**

Ln 85, delete “2000”

**Done.**

Ln 88, how many sub-samples (disks) from each leaf?

**We now clarify that “Each surface-sterilized leaf was subsampled once…”**

Ln 92, “very few” is too subjective, “less than xx” would be better

**The statement now reads “After 3 weeks, fewer than 10 total fungal colonies grew out from the entire collection of leaf disks.”**

Ln 92-96, I suppose the plates were inspected every day to isolate strains before R selected fungi (e.g. Penicillium, Trichoderma,…) form and release conidia

**Yes**.

Ln 103, “ITS is considered a universal barcode for fungi” delete this sentence because superfluous

**Deleted**

Ln 116, 119,…, “sp.” must not be italicized; What is “Ophiognomonia 1”? perhaps “Ophiognomonia sp.”?

**These have been corrected throughout the manuscript.**

Ln 132, for a total of 640 plates, is it correct?

**Yes, this fact has been added.**

Ln 133-136, it is not clear which is the size of the plates.

**We now specify that the plates were 5 cm diameter.**

I do not think that 200 ml of medium was added to each plate. Moreover the amount of each component of the medium should be reported as weight (g, mg,…) per L.

**The components of the media are now given per liter.**

Ln 137, was the leaf litter autoclaved?

**Yes, the leaf litter was added to the medium prior to autoclaving. We now write “All media components were added prior to autoclaving.”**

Ln 143, how did you separate mycelium biomass from residues of litter?

**Surprisingly, the vast majority of the ground leaf litter was easily separated from the mycelium during the boiling process.**

**Do you want your identity to be public for this peer review?** For information about this choice, including consent withdrawal, please see our [Privacy Policy](https://www.plos.org/privacy-policy).

Reviewer #1: No

Reviewer #2: No
